# Supplementary material for: The UFM1 system regulates ER-phagy through the ufmylation of CYB5R3
Source: Nat Commun. 2022 Dec 21;13:7857. doi: 10.1038/s41467-022-35501-0 (PMC9772183; doi:10.1038/s41467-022-35501-0)

Supplementary Information

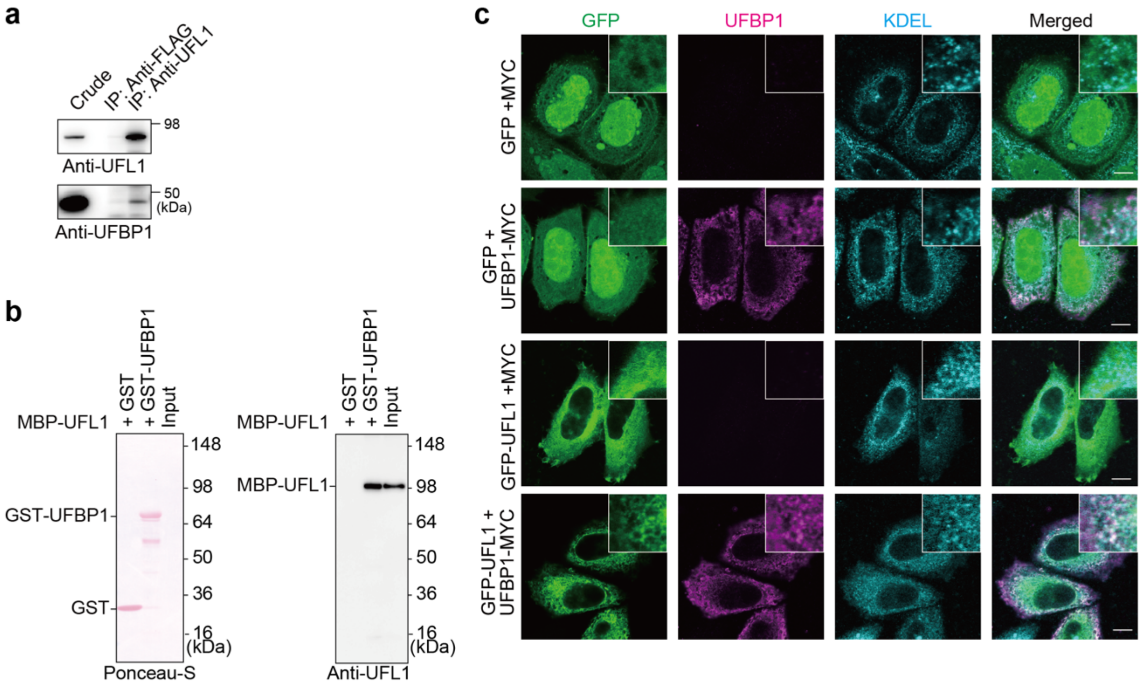

Supplementary Figure S1 UFL1 forms a stable complex with UFBP1 on the ER

(a) Immunoprecipitation assay. Lysates prepared from HEK293T cells were immunoprecipitated with anti-UFL1 antibody followed by immunoblot analyses with the indicated antibodies. Data shown are representative of three separate experiments.

(b) *In vitro* pull-down assay. Recombinant GST-tagged UFBP1 was mixed with recombinant maltose-binding protein (MBP)-tagged UFL1. MBP-UFL1 binding to GST-UFBP1 was estimated by immunoblot analysis. Data shown are representative of three independent experiments.

(c) Immunofluorescence analysis. GFP-tagged UFL1 was co-transfected with MYC-tagged UFBP1 (UFBP1-MYC) into *UFBP1*-deficient HeLa cells. Twenty-four hours after transfection, cells were immunostained with anti-UFBP1 and anti-KDEL antibodies. Bars: 20 µm. Data shown are representative of three independent experiments.

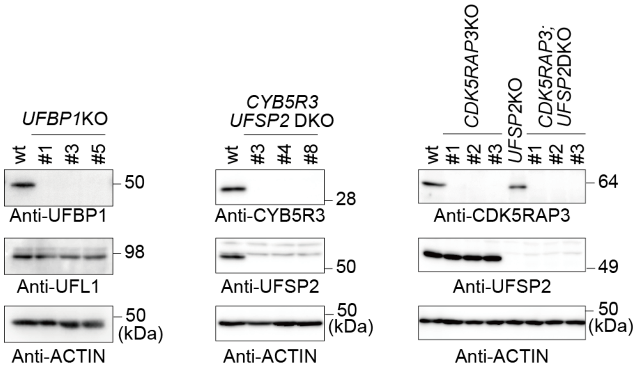

Supplementary Figure S2 Generation of knockout cell lines

Immunoblot analysis. The indicated genotype cells were lysed, then subjected to SDS-PAGE followed by immunoblot analysis with the indicated antibodies. Data shown are representative of three independent experiments.

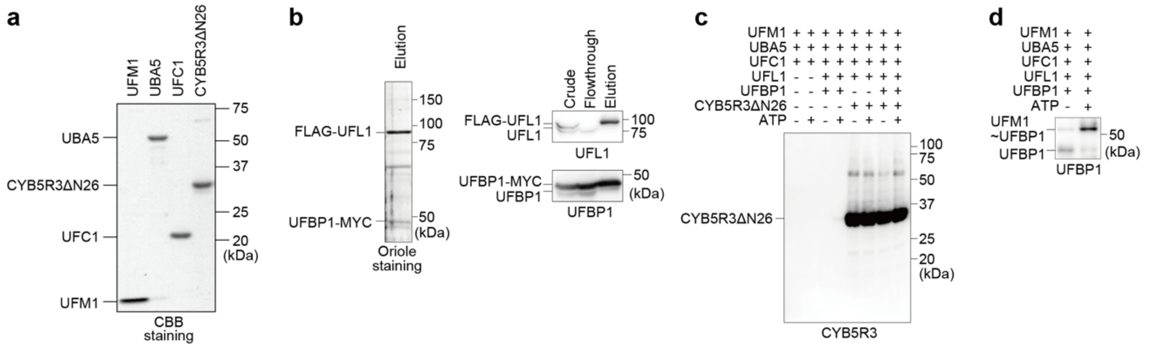

Supplementary Figure S3 ***In vitro*** **ufmylation assay**

(a) CBB staining. Recombinant UFM1, UBA5, UFC1, and CYB5R3ΔN26 purified from *E. coli* were subjected to SDS-PAGE followed by CBB staining. Data shown are representative of three independent experiments.

(b) Oriole staining and immunoblot analysis. FLAG-UFL1 and UFBP1-MYC were co-expressed in *UFC1*-knockout HEK293T cells. The cell lysates were immunoprecipitated with anti-DDDDK–tagged pAb-agarose, and the immunoprecipitants were eluted by FLAG peptide and subjected to SDS-PAGE followed by oriole staining (left panel). The cell lysates (crude) as well as flow-through and elution fractions in the immunoprecipitation were subjected to SDS-PAGE followed by immunoblot analysis with the indicated antibodies (right panels). Data shown are representative of three independent experiments.

(c) UFM1, UBA5, UFC1, and CYB5R3ΔN26 described in (a) and FLAG-UFL1 and UFBP1-MYC shown in (b) were incubated in the presence or absence of ATP for 90 min, and the mixture was subjected to SDS-PAGE followed by immunoblot analysis with anti-CYB5R3 antibody. Data shown are representative of three separate experiments.

(d) *In vitro* ufmylation assay. Recombinant UFM1, UBA5, and UFC1 were incubated with FLAG-UFL1 and UFBP1-MYC prepared from *UFC1*-deficient HEK293T cells in the presence or absence of ATP for 90 min, and the mixture was subjected to SDS-PAGE followed by immunoblot analysis with anti-UFBP1 antibody. Data shown are representative of three separate experiments.

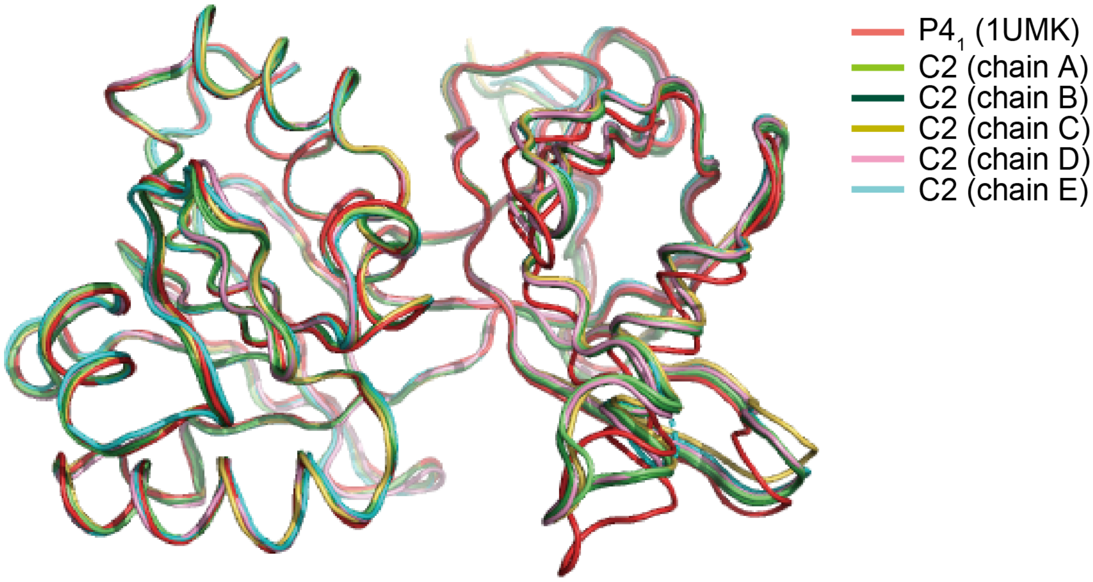

Supplementary Figure S4 Structure of human CYB5R3

Structural comparison of five copies (chains A to E) of human CYB5R3 in the C2 crystal with that in the $P4_1$ crystal (1UMK). Main-chain atoms of the NADH domain were superimposed by minimizing the rms difference.

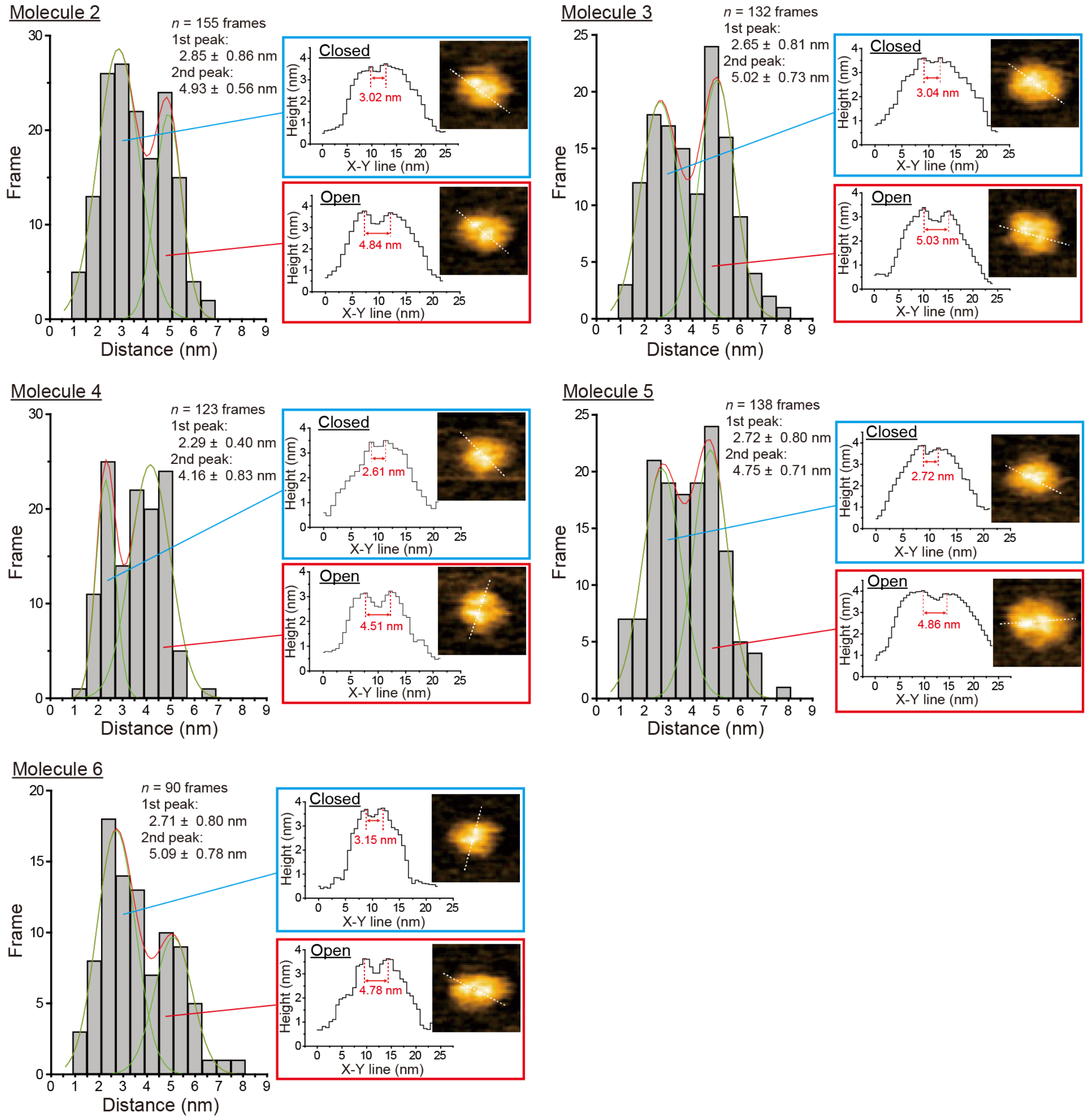

Supplementary Figure S5 Successive HS-AFM images of CYB5R3

Histograms of the distances between two globular lobes obtained from 5 molecules. Representative images of closed and open conformation with height profiles are shown right.

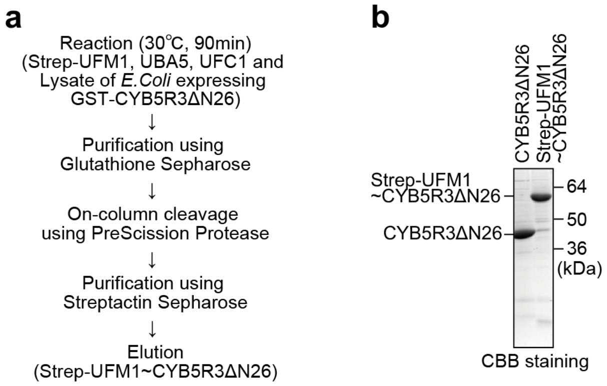

Supplementary Figure S6 Purification of ufmylated CYB5R3

(a) Procedure for purification of UFM1-conjugated CYB5R3ΔN26. Strep-UFM1ΔC2 (9 μM), UBA5 (2 μM), and UFC1 (5 μM) were mixed with lysate of *E.coli* expressing GST-CYB5R3ΔN26 in a reaction buffer containing 2 mM ATP and 10 mM $MgCl_2$. The mixtures were incubated at 30°C for 90 min. GST-CYB5R3ΔN26 conjugated with Strep-UFM1 was purified with Glutathione Sepharose, and the GST-tag was cleaved using PreScission Protease. Thereafter, UFM1-conjugated CYB5R3ΔN26 was purified with Streptactin Sepharose, and free GST-CYB5R3ΔN26 was removed.

(b) CBB staining of purified free CYB5R3ΔN26 and UFM1-conjugated CYB5R3ΔN26. Data shown are representative of three independent experiments.

a

b

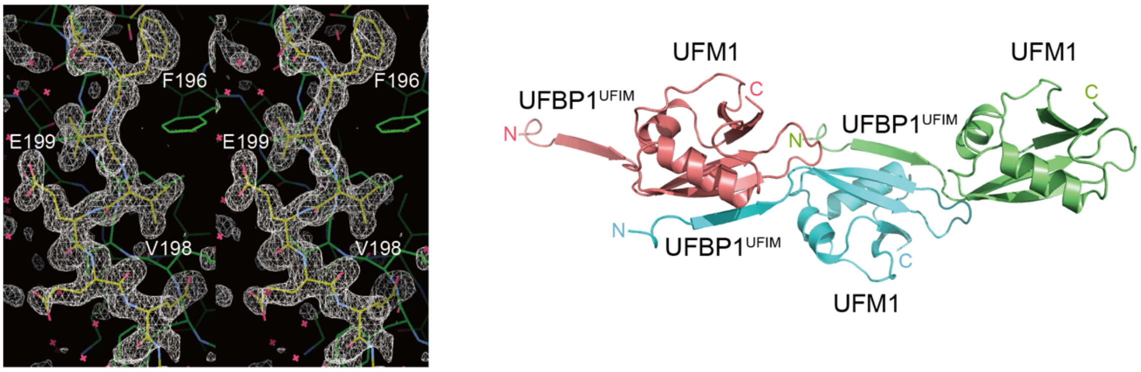

Supplementary Figure S7 Crystallographic analysis of UFM1 in a complex with the UFIM of UFBP1

(a) Stereoscopic view of the annealed omit map (Fo-Fc at 2.5σ) of the UFIM (residues 195-200) of UFBP1. The Strick model shows the final structure of the UFM1-UFBP1 UFIM complex, where carbon atoms of UFIM and UFM1 are colored yellow and green, respectively.

(b) Head-to-tail interaction of UFBP1 UFIM-UFM1 fusion proteins in the crystal. N and C indicate the N-terminus and C-terminus of the fusion protein, respectively.

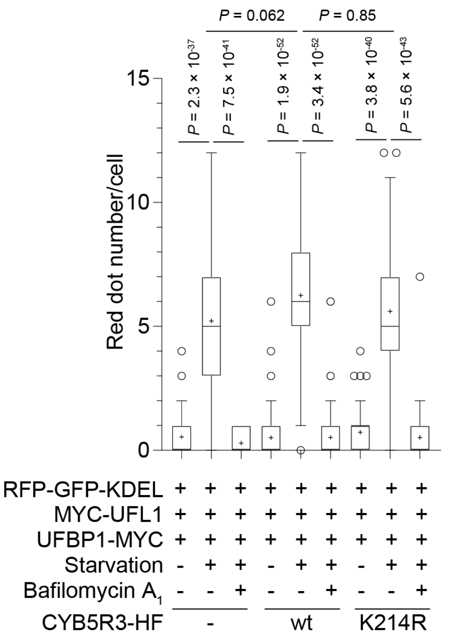

Supplementary Figure S8 ER-phagy assay with ssRFP-GFP-KDEL

ssRFP-GFP-KDEL together with MYC-UFL1, UFBP1-MYC and CYB5R3-His-FLAG (CYB5R3-HF) or CYB5R3^{K214R}-HF were co-transfected into *CYB5R3*-deficient HeLa cells. Forty-eight hours after transfection, the cells were cultured under nutrient-rich or deprived conditions for 9 h in the presence or absence of Bafilomycin A_1. The cells were fixed and observed by confocal microscopy. The number of single RFP-positive punctae per cell was determined using a Benchtop High-Content Analysis System and CellPathfinder software without bias. The numbers of cells used to count the RFP-positive punctae were 60 cells in each experimental settings. Data are means ± s.e. Statistical analysis was performed by Šidák's multiple comparison test after one-way ANOVA.

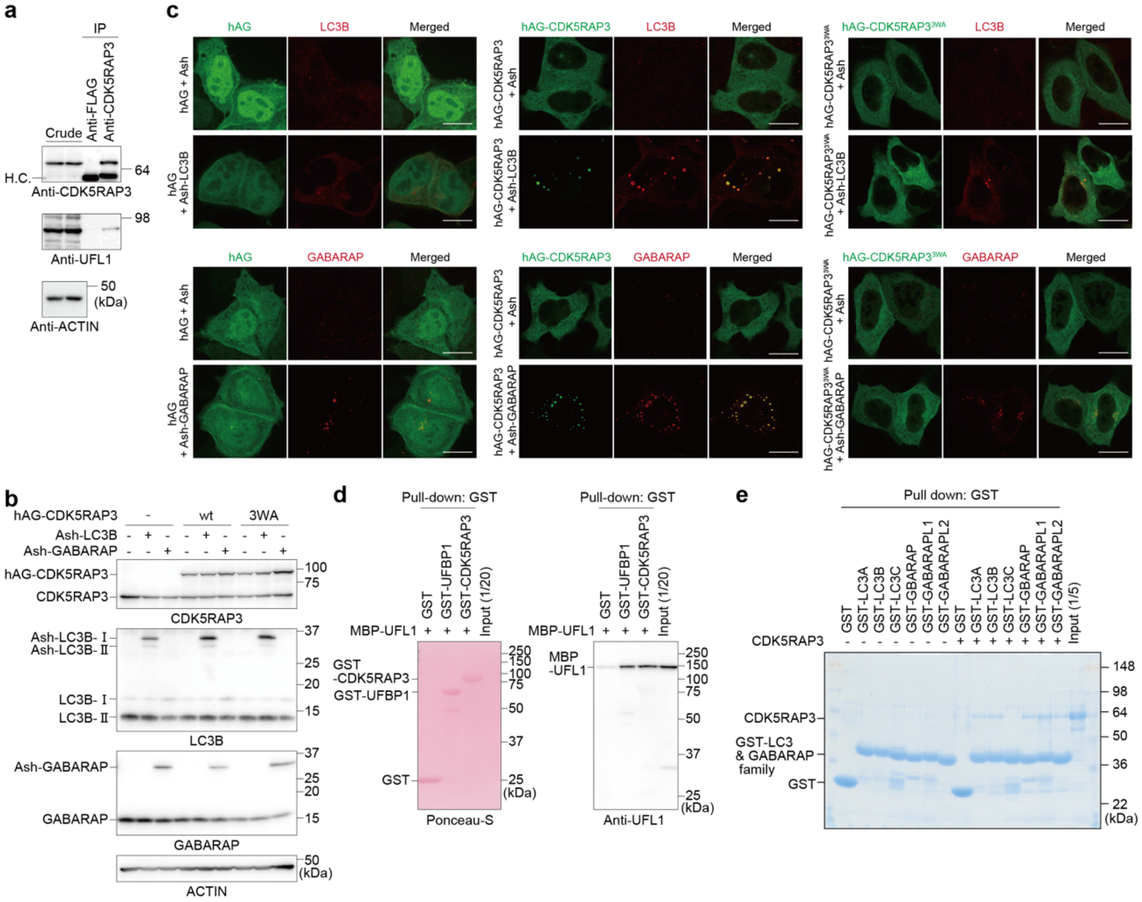

Supplementary Figure S9 The interaction of CDK5RAP3 with UFL1 and ATG8-family proteins

(a) Immunoprecipitation assay. Lysates prepared from HEK293T cells were immunoprecipitated with anti-CDK5RAP3 antibody followed by immunoblot analyses with the indicated antibodies. Data shown are representative of three separate experiments.

(b) Immunoblot analysis. hAG-tagged CDK5RAP3 or its LIR mutant CDK5RAP3^{3WA} and Ash-LC3B or GABARAP were co-expressed in HeLa cells. Forty-eight hours after transfection, the cell lysates were subjected to SDS-PAGE, followed by immunoblot analysis. Data shown are representative of three separate experiments.

(c) Fluoppi assay. HeLa cells transiently expressing hAG-tagged CDK5RAP3 or its LIR mutant CDK5RAP3^{3WA} and Ash-LC3B or GABARAP were immunostained. Scale bars, 20 µm. Data shown are representative of three independent experiments.

(d) *In vitro* pull-down assay. Recombinant GST-tagged UFBP1ΔN50 or CDK5RAP3 were mixed with recombinant maltose-binding protein (MBP)-tagged UFL1. MBP-UFL1 binding to GST-UFBP1ΔN50 or CDK5RAP3 was estimated by immunoblot analysis. Data shown are representative of three independent experiments. Data shown are representative of four independent experiments.

(e) *In vitro* pull-down assay. GST-tagged LC3- or GABARAP-family proteins were mixed with CDK5RAP3. CDK5RAP3 binding to GST-LC3- or GABARAP-family proteins was estimated by Coomassie brilliant blue staining. Data shown are representative of three independent experiments.

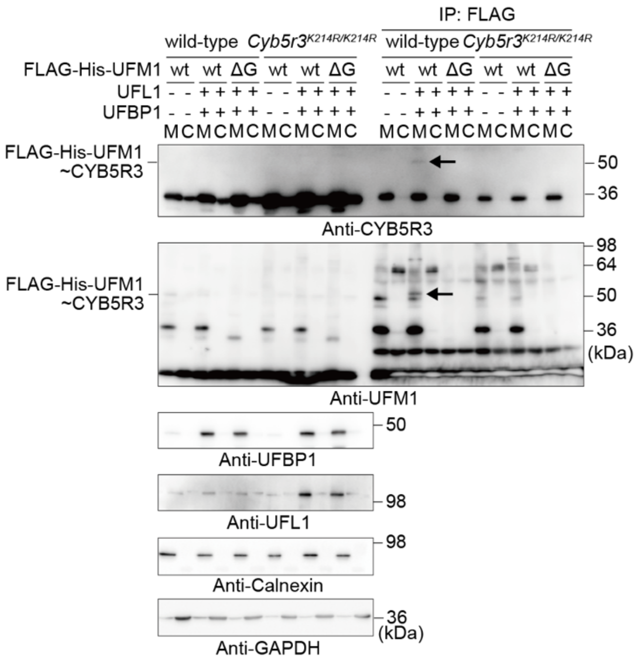

Supplementary Figure S10 Defective ufmylation of Cyb5r3 in $Cyb5r3^{K214R/K214R}$ cells

Immunoblot analysis. Wild-type or $Cyb5r3^{K214R/K214R}$ mouse embryonic fibroblasts were infected with the indicated adenovirus vectors. Forty-eight hours after infection, cells were fractionated into microsomal (M) and cytoplasmic (C) fractions. After denaturing with 1% SDS containing buffer, the fractions were diluted with SDS-minus buffer (final SDS concentration 0.1%) and then immunoprecipitated with anti-CYB5R3 antibody followed by immunoblot analyses with anti-CYB5R3 and anti-UFM1 antibodies. Data shown are representative of three separate experiments.

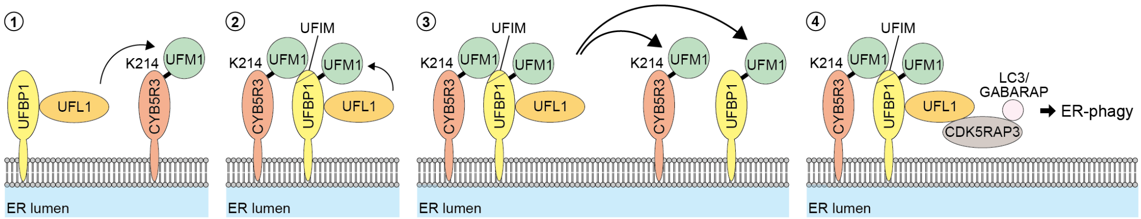

Supplementary Figure S11 Model of ER-phagy mediated by CYB5R3 ufmylation

First, CYB5R3 is ufmylated on the ER, and the ufmylated CYB5R3 is therefore recognized by UFBP1. Second, this interaction promotes UFBP1 ufmylation and increases the E3-ligating activity of UFL1-UFBP1 against CYB5R3. Third, UFL1 in complex with ufmylated CYB5R3 and UFBP1 interacts with CDK5RAP3, leading to autophagic degradation of ER subdomains.

Uncropped blot images for supplementary figures

Supplementary Figure S1a

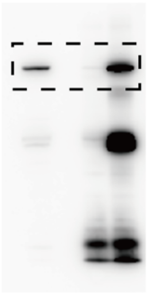

Anti-UFL1

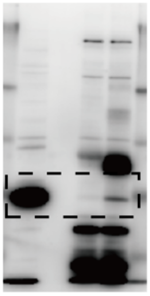

Anti-UFBP1

Supplementary Figure S2

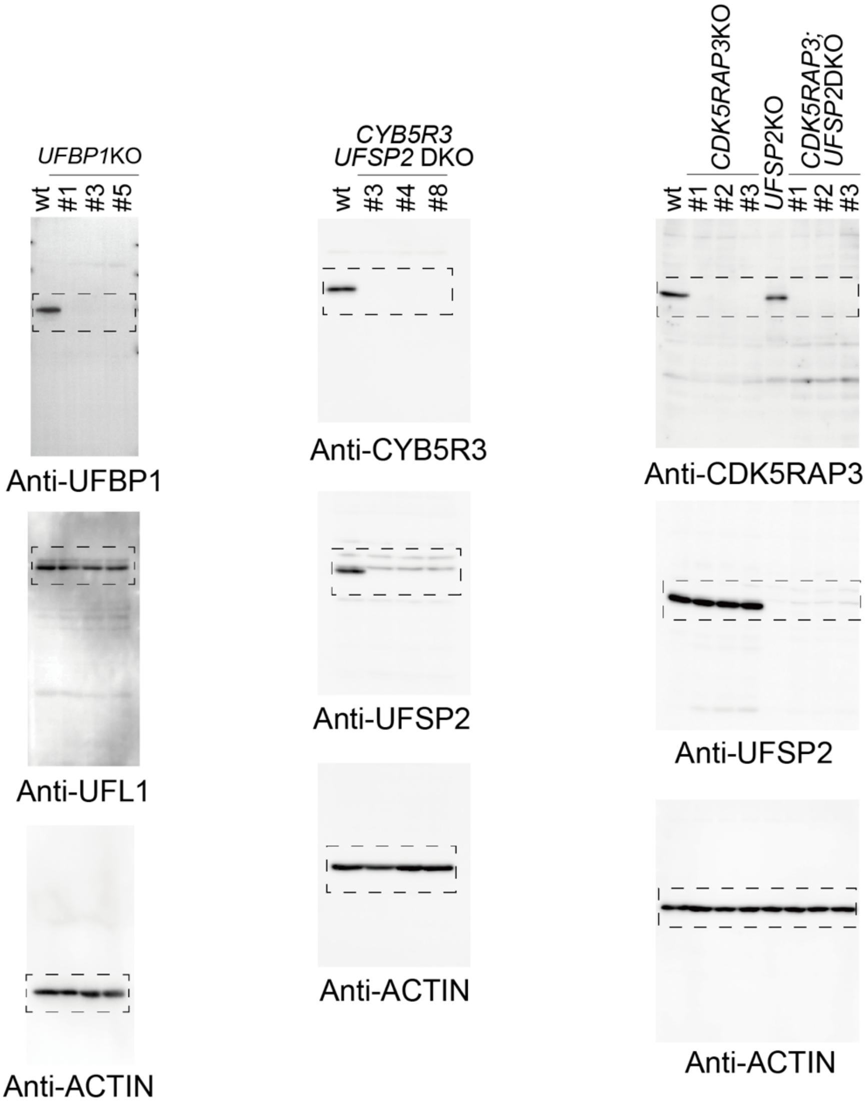

Supplementary Figure S3b

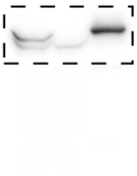

Anti-UFL1

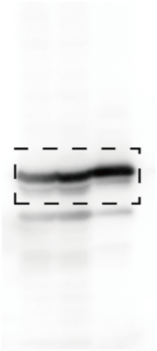

Anti-UFBP1

Supplementary Figure S3d

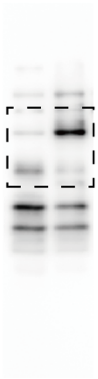

Anti-UFBP1

Supplementary Figure S9a

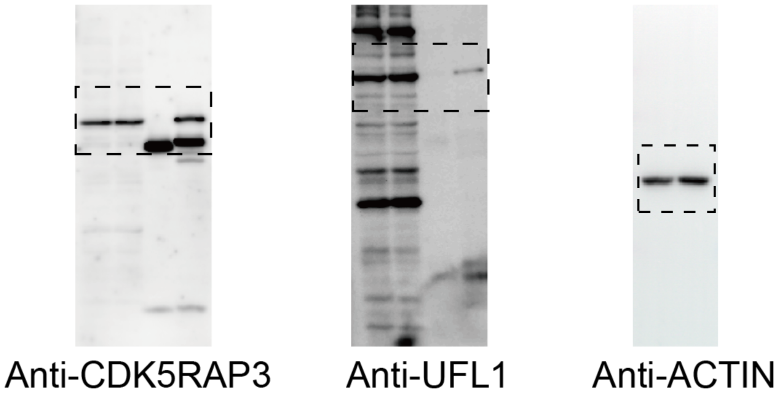

Supplementary Figure S9b

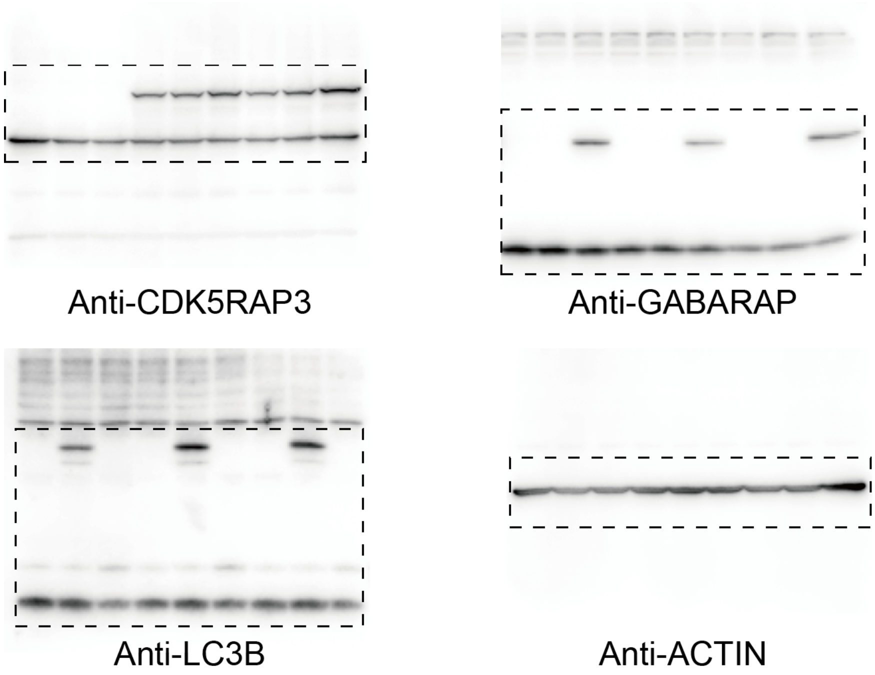

Supplementary Figure S10

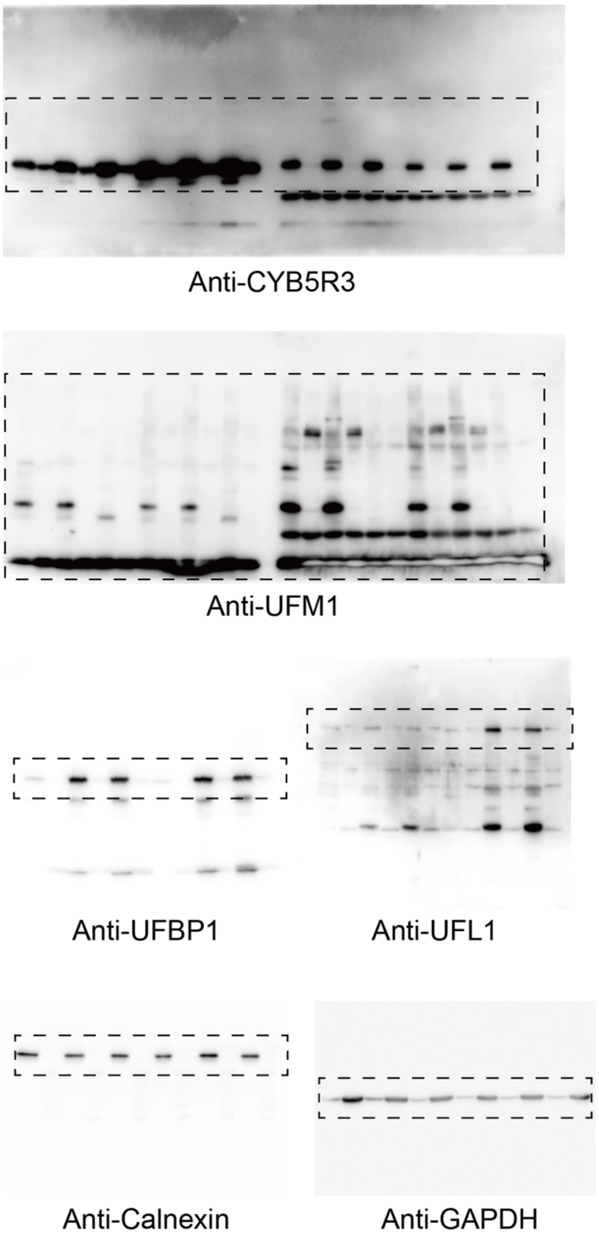

Supplement: Supplementary file 1 — Supplementary Information [file 41467_2022_35501_MOESM1_ESM.pdf]
