# Supplementary material for: The UFM1 system regulates ER-phagy through the ufmylation of CYB5R3
Source: Nat Commun. 2022 Dec 21;13:7857. doi: 10.1038/s41467-022-35501-0 (PMC9772183; doi:10.1038/s41467-022-35501-0)
Supplement: Supplementary file 3 — Description of Additional Supplementary Files [file 41467_2022_35501_MOESM3_ESM.pdf]

File name: Supplementary Data 1

Description: Identification of UFM1-binding proteins under denaturing conditions by mass spectrometric analysis.

Among the proteins identified with >99% confidence, those exclusively found in HEK293T cells expressing FLAG-His-UFM1ΔC2 but not FLAG-His-UFM1ΔC3 were defined as UFM1- interacting proteins. Based on this criterion, we reasoned that 60 of 151 proteins originally identified in the co-immunoprecipitation assay using FLAG-His-UFM1ΔC2 were UFM1- interacting proteins. %Cov (95), % of matching amino acids in peptides identified with >95% confidence per the total number of amino acids in the protein sequence; Peptides (95%), the number of distinct peptides identified with at least 95% confidence; GN, gene name; AcNo., ID in UniProtKB.

File name: Supplementary Data 2

Description: Identification of UFM1-binding proteins, specifically under E3-expressing conditions, by mass spectrometric analysis.

To identify UFM1-binding proteins under expression of UFL1 and UFBP1, FLAG-UFM1 alone or in various combinations with MYC-UFL1 and UFBP1-MYC was expressed in HEK293T cells. As a control, FLAG vector (mock) was also transfected in HEK293T cells. These cell lysates were subjected the immunoprecipitates to mass spectrometry (MS) (n = 4). Among the identified proteins, we extracted those that were not identified in mock MS and those that were also identified in mock MS but whose iBAQ values were less than one-fifth of the other samples. The candidate proteins were further narrowed down by selecting those that were detected more than once only when both MYC-UFL1 and UFBP1-MYC were expressed.

File name: Supplementary Data 3

Description: The amount of saturated, monounsaturated and polyunsaturated phosphatidylcholine (PC) and phosphatidylethanolamine (PE).

UFL1, UFBP1 and CYB5R3 (wild-type) or CYB5R3K214R (KR mutant) were transfected into CYB5R3 UFSP2 double-knockout HEK293T cells. 48 h after the transfection, the cells were harvested and subjected to lipid extraction. A portion of the extracted lipid was injected onto the LC-MS/MS to measure fatty acid molecular species of phosphatidylcholine (PC) and phosphatidylethanolamine (PE).

File name: Supplementary Movie 1

Description: HS-AFM movie of CYB5R3ΔN26

The images were acquired at 5 frames/s. Scale: 60 nm × 60 nm (100 pixel × 100 pixel), height scale: 0–4 nm.
