# Supplementary material for: The UFM1 system regulates ER-phagy through the ufmylation of CYB5R3
Source: Nat Commun. 2022 Dec 21;13:7857. doi: 10.1038/s41467-022-35501-0 (PMC9772183; doi:10.1038/s41467-022-35501-0)
Supplement: Supplementary file 8 — Reporting Summary [file 41467_2022_35501_MOESM8_ESM.pdf]

nature portfolio

Corresponding author(s): Masaaki Komatsu

Last updated by author(s): Nov 29, 2022

Reporting Summary

Nature Portfolio wishes to improve the reproducibility of the work that we publish. This form provides structure for consistency and transparency in reporting. For further information on Nature Portfolio policies, see our Editorial Policies and the Editorial Policy Checklist.

Statistics

For all statistical analyses, confirm that the following items are present in the figure legend, table legend, main text, or Methods section.

n/a	Confirmed	
☐	☒	The exact sample size (*n*) for each experimental group/condition, given as a discrete number and unit of measurement
☐	☒	A statement on whether measurements were taken from distinct samples or whether the same sample was measured repeatedly
☐	☒	The statistical test(s) used AND whether they are one- or two-sided<br>*Only common tests should be described solely by name; describe more complex techniques in the Methods section.*
☒	☐	A description of all covariates tested
☐	☒	A description of any assumptions or corrections, such as tests of normality and adjustment for multiple comparisons
☐	☒	A full description of the statistical parameters including central tendency (e.g. means) or other basic estimates (e.g. regression coefficient) AND variation (e.g. standard deviation) or associated estimates of uncertainty (e.g. confidence intervals)
☐	☒	For null hypothesis testing, the test statistic (e.g. *F*, *t*, *r*) with confidence intervals, effect sizes, degrees of freedom and *P* value noted<br>*Give P values as exact values whenever suitable.*
☒	☐	For Bayesian analysis, information on the choice of priors and Markov chain Monte Carlo settings
☒	☐	For hierarchical and complex designs, identification of the appropriate level for tests and full reporting of outcomes
☒	☐	Estimates of effect sizes (e.g. Cohen's *d*, Pearson's *r*), indicating how they were calculated

Our web collection on statistics for biologists contains articles on many of the points above.

Software and code

Policy information about availability of computer code

Data collection	The data were collected using Multi Gauge (3.2) and Benchtop High-Content Analysis System (CQ1, Yokogawa Electric Corp)
Data analysis	CellPathfinder (3.06.01.08), XDS (ver. May 1, 2016), MolRep (11.0), Phenix (ver. 1.11.1), COOT (ver. 0.8.9), Kodec (4.4.7.39), ProteinPilot (v5.0.1), Multi Gauge (3.2), GraphPad Prism (9.2.0), Adobe Photoshop (22.5.9)

For manuscripts utilizing custom algorithms or software that are central to the research but not yet described in published literature, software must be made available to editors and reviewers. We strongly encourage code deposition in a community repository (e.g. GitHub). See the Nature Portfolio guidelines for submitting code & software for further information.

Data

Policy information about availability of data

All manuscripts must include a data availability statement. This statement should provide the following information, where applicable:

- Accession codes, unique identifiers, or web links for publicly available datasets
- A description of any restrictions on data availability
- For clinical datasets or third party data, please ensure that the statement adheres to our policy

The proteomics data generated in this study have been deposited in the ProteomeXchange under accession code PXD021225 [http://proteomecentral.proteomexchange.org/cgi/GetDataset?ID=PXD021225] and PXD038409 [http://proteomecentral.proteomexchange.org/cgi/GetDataset?ID=PXD038409]. Coordinates and structure factors of UFBP1 UFIM-UFM1 fusion and CYB5R3 have been deposited in the Protein Data Bank under accession codes

7W3N [https://www.rcsb.org/structure/7W3N] and 7W3O [https://www.rcsb.org/structure/7W3O], respectively. All figures and movies are available in figshare [https://doi.org/10.6084/m9.figshare.21641051]. Source data are provided with this paper.

Human research participants

Policy information about studies involving human research participants and Sex and Gender in Research.

Reporting on sex and gender	n/a
Population characteristics	n/a
Recruitment	n/a
Ethics oversight	n/a

Note that full information on the approval of the study protocol must also be provided in the manuscript.

Field-specific reporting

Please select the one below that is the best fit for your research. If you are not sure, read the appropriate sections before making your selection.

☒ Life sciences ☐ Behavioural & social sciences ☐ Ecological, evolutionary & environmental sciences

For a reference copy of the document with all sections, see nature.com/documents/nr-reporting-summary-flat.pdf

Life sciences study design

All studies must disclose on these points even when the disclosure is negative.

Sample size	Sample size calculation was not performed prior to the experiment. The sample size was determined based on experience of similar assays performed earlier. All experiments were repeated at least three times independently and s.e.m. were calculated from those data.
Data exclusions	No data were excluded.
Replication	All experiments were repeated at least three times independently. Replicated experiments are explained in the text.
Randomization	No randomization was applicable since there was no organization in experimental groups.
Blinding	Investigators were blinded to group allocation during experiments. Data reported for experiments are not subjective.

Reporting for specific materials, systems and methods

We require information from authors about some types of materials, experimental systems and methods used in many studies. Here, indicate whether each material, system or method listed is relevant to your study. If you are not sure if a list item applies to your research, read the appropriate section before selecting a response.

Materials & experimental systems

n/a	Involved in the study	
☐	☒	Antibodies
☐	☒	Eukaryotic cell lines
☒	☐	Palaeontology and archaeology
☐	☒	Animals and other organisms
☒	☐	Clinical data
☒	☐	Dual use research of concern

Methods

n/a	Involved in the study	
☒	☐	ChIP-seq
☒	☐	Flow cytometry
☒	☐	MRI-based neuroimaging

Antibodies

Antibodies used	For primary antibodies<br>UFM1 (ab109305, Abcam, Cambridge, UK; 1:1000)<br>UFSP2 (ab185965, Abcam; 1:1000)<br>CYB5R3 (GTX84646; GeneTex, Irvine, CA, USA; 1:1000)<br>CYB5R3 (10894-1-AP; Proteintech, Rosemont, IL, USA; 1:1000)

UFBP1 (21445-1-AP, Proteintech; 1:1000)
UFL1 (A303-456A; Bethyl Laboratories, Montgomery, TX, USA; 1:1000)
CDK5RAP (H00080279-M01; Novus Biologicals, Englewood, CO, USA; 1:500)
Calnexin (sc-46669; Santa Cruz Biotechnology. Dallas, TX, USA; 1:500)
GAPDH (MAB374, Merck Millipore; 1:1000)
PRL26 (ab59567, Abcam; 1:1000)
ACTIN (A1978; Sigma-Aldrich, Burlington, MO, USA; 1:2000)
MYC (M192-3, Medical & Biological Laboratories, Nagoya, Japan; 1:1000)
FLAG (M185-3L, Medical & Biological Laboratories; 1:2000)
KDEL (ADI-SPA-827-D, Enzo Life Sciences, Farmingdale, NY, USA; 1:200)
PDI (sc-20132, Santa Cruz Biotechnology; 1:200)
FIP200 (17250-1-AP, Proteintech; 1:200)
WIPI2 (ab105459, Abcam; 1:200)
LC3 (PM036, Medical & Biological Laboratories; 1:200)
GABARAP (PM037, Medical & Biological Laboratories; 1:200)

For secondary antibodies:
horseradish peroxidase-conjugated goat anti-mouse IgG (H+L) (115-035-166, Jackson ImmunoResearch Laboratories, Inc.; 1:10000)
horseradish peroxidase-conjugated goat anti-rabbit IgG (H+L) (111-035-144, Jackson ImmunoResearch Laboratories, Inc.; 1:10000)
Goat Anti-Rabbit IgG (H+L) Cross-Adsorbed Secondary Antibody, Alexa Fluor 568 (A11036, Thermo Fisher Scientific; 1:1000)
Goat Anti-Mouse IgG (H+L) Highly Cross-Adsorbed Secondary Antibody, Alexa Fluor 647 (A21236, Thermo Fisher Scientific; 1:1000)

Validation

UFM1 (ab109305, Abcam; https://www.abcam.co.jp/ufm1-antibody-epr42642-ab109305.html)
UFSP2 (ab185965, Abcam; https://www.abcam.co.jp/ufsp2-antibody-epr13424-ab185965.html)
CYB5R3 (GTX84646, GeneTex; https://www.genetex.com/Product/Detail/CYB5R3-antibody-2A10/GTX84646)
CYB5R3 (10894-1-AP, Proteintech; https://www.ptglab.co.jp/products/CYB5R3-Antibody-10894-1-AP.htm)
UFBP1 (21445-1-AP, Proteintech; https://www.ptglab.co.jp/products/DDRGK1-Antibody-21445-1-AP.htm)
UFL1 (A303-456A; Bethyl Laboratories; https://www.thermofisher.com/antibody/product/UFL1-Antibody-Polyclonal/A303-456A)
CDK5RAP (H00080279-M01; Novus Biologicals; https://www.novusbio.com/primary-antibodies/cdk5rap3)
Calnexin (sc-46669; Santa Cruz Biotechnology; https://www.scbt.com/p/calnexin-antibody-e-10)
GAPDH (MAB374, Merck Millipore; https://www.merckmillipore.com/JP/ja/product/Anti-Glyceraldehyde-3-Phosphate-Dehydrogenase-Antibody-clone-6C5,MM_NF-MAB374)
PRL26 (ab59567, Abcam; 1:2000)
ACTIN (A1978; Sigma-Aldrich; https://www.sigmaaldrich.com/JP/ja/search/a1978?focus=products&page=1&perpage=30&sort=relevance&term=a1978&type=product_name)
MYC (M192-3, Medical & Biological Laboratories; https://www.mblintl.com/products/m192-3/)
FLAG (M185-3L, Medical & Biological Laboratories; https://www.mblbio.com/bio/g/dtl/A/index.html?pcd=M185-3L)
KDEL (ADI-SPA-827-D, Enzo Life Sciences; https://www.enzolifesciences.com/ADI-SPA-827/kdel-monoclonal-antibody-10c3/)
PDI (sc-20132, Santa Cruz Biotechnology; https://www.scbt.com/p/pdi-antibody-h-160)
FIP200 (17250-1-AP, Proteintech; https://www.ptglab.co.jp/products/RB1CC1-Antibody-17250-1-AP.htm)
WIPI2 (ab105459, Abcam; https://www.abcam.co.jp/wipi2-antibody-2a2-ab105459.html)
LC3 (PM036, Medical & Biological Laboratories; https://www.mblintl.com/products/pm036/)
GABARAP (PM037, Medical & Biological Laboratories; https://ruo.mbl.co.jp/bio/dtl/A/?pcd=PM037)

Eukaryotic cell lines

Policy information about cell lines and Sex and Gender in Research

Cell line source(s)

HEK293T (ATCC CRL-3216) and HeLa (ATCC CCL2) cell lines were obtained from ATCC.

Authentication

All cell lines were authenticated by STR profile.

Mycoplasma contamination

The mammalian cell lines were confirmed to be negative for mycoplasma contamination by observation by fluorescence microscopy.

Commonly misidentified lines
(See ICLAC register)

No commonly misidentified cell lines were used.

Animals and other research organisms

Policy information about studies involving animals; ARRIVE guidelines recommended for reporting animal research, and Sex and Gender in Research

Laboratory animals

To induce CRISPR/Cas-mediated Cyb5r3 K214R knock-in, approximately CRISPR/Cas9 solution was injected into the oviductal lumens of female C57BL/6N mice at day 0.7 of pregnancy. Description of research mice used for experiments can be found in the relevant legends and methods. 4-5 months male and female wild-type, Cyb5r3K214R/+ and Cyb5r3K214R/K214R mice were used. All mice housed in a specific pathogen-free room under temperature (23 ± 3 degrees), and humidity (40~60%) controlled conditions with 12/12h light dark cycle.

Wild animals

Wild-type mice were not used in this study.

Reporting on sex

The animal data generated here was analysed not considering sex as a variable.

Field-collected samples	This study did not involve field collected samples.
Ethics oversight	Mice were housed in specific pathogen-free facilities, and the Ethics Review Committee for Animal Experimentation of Juntendo University approved the experimental protocol (2022226).

Note that full information on the approval of the study protocol must also be provided in the manuscript.
